# Genomic Diversity of the Retinta Breed Derived from Two Ancestral Bovine Lineages

**DOI:** 10.3390/vetsci11060247

**Published:** 2024-05-30

**Authors:** Gabriel Anaya, Rosa Morales, Sebastián Demyda-Peyrás, Samuel Moreno-Jiménez, José María Jiménez, Antonio Molina

**Affiliations:** 1 Department of Genetics, University of Córdoba, CN IV, KM 396, 17071 Córdoba, Spain; b22ancag@uco.es (G.A.); v22mocir@uco.es (R.M.);; 2 Agricultural and Livestock Experimental Center (CEAG), The Council of Cadiz, 11400 Jerez de la Frontera, Spain

**Keywords:** cattle, Retinta, genetic lines, strains, genomic, genealogical

## Abstract

**Simple Summary:**

The Retinta cattle breed from Spain is derived from two ancestral bovine lineages, which are clearly characterized based on coat color: blond and brown/red. Currently, despite a great level of admixture, two pure genetic lines remain (Retinto Extremeño and Andalusian Blond). This study analyzed the genealogy and genetics of over 22,000 animals, revealing high genetic diversity overall, but lower diversity within each subpopulation, with the Retinto line having the greatest influence on the breed. A special subgroup within the Andalusian Blond lineage, known as the Tamarona cow, was identified as genetically unique and should be protected due to its small population size. Preserving genetic diversity within the Retinta breed is crucial to ensure sustainable breeding in the breed’s natural environment.

**Abstract:**

The Retinta breed, an autochthonous type of Spanish beef cattle, is highly adapted to breeding in its natural environment, which is characterized by a Mediterranean climate. The origins of this breed can be traced to two ancestral bovine stocks, which gave rise to distinct morphotypes differentiated primarily by coat color, alongside other significant traits such as growth, morphological conformation and temperament. Specifically, one morphotype comprises blond animals (Rubia Andaluza), genetically resembling the ancestral Bos taurus Aquitanicus, while the other encompasses brown- and red-colored animals (Retinta Extremeña) originating from Bos taurus Turdenatus stock. Over decades, these populations have undergone hybridization, leading to a unified population, albeit with the original subpopulations largely maintaining their genetic integrity. The objective of this study was to undertake genealogical and genomic characterization of these genetic lines, including a particular subpopulation within the blond animals (Tamarona cow). To achieve this, the genealogical records of 22,004 active animals were analyzed, and over 63,000 SNPs from a total of 1030 animals were examined for genomic characterization. Genealogical analysis revealed pedigree completeness and a high level of effective population size (Ne) across the entire population, yet relatively low Ne values within each pure line (ranging from 28.38 to 34.47). These findings underscore the ongoing efforts of the National Association of Retinta Breeders (ACRE) over the past decades to mitigate the loss of variability in this breed. The genomic characterization highlights the persistent differences within the original population and the predominant influence of the Retinto line within the current breed, as evidenced by principal component analysis (PCA) and admixture analysis. Furthermore, the identification of the Tamarona subpopulation within the blond lineage underlines its unique genetic composition, warranting its recognition as an official genetic line within the current Retinta breed. Given the small population size of these lines, particularly the Tamarona subpopulation, protective measures are imperative to preserve this distinct gene pool. Such measures would enhance the genetic diversity of the Retinta breed, which is essential for sustainable breeding practices in its natural habitats.

## 1. Introduction

Retinta is an autochthonous Spanish cattle breed widely raised in the south and west of Spain, where approximately 90% of all specimens in this country are located. However, the breed is also present in other countries, such as Portugal, Argentina and Brazil [1]. According to data from the Spanish Ministry of Agriculture, Fisheries and Food, in 2022, 22,963 animals were registered in the herd book administered by the National Association of Retinto Breeders (ACRE), of which 16,448 were cows and 307 were bulls [2].

The Retinta is bred in an extensive management system in a special habitat known as dehesa, which includes mountain ranges covered by Mediterranean shrubs (*Cistus* spp., *Erica* spp., *Arbutus unedo*, *Phyllirea* spp., *Genista hirsuta*, *Lavandula* spp.) and trees (*Quercus* spp., *Olea europaea*), along with lower, flatter lands and open oak woodlands [3]. As an autochthonous breed, these animals can survive the summer in these semiarid, dry and hot areas with scarce resources better than other breeds or crossbred animals [4]. The Retinta plays an important role in this natural environment by supporting the biodiversity of the dehesa thanks to its low impact on the land, normally in farms near to National Parks, which also enables the rural human population that protects these highly-valued, vital ecosystems to make a living.

In addition to its current productive importance and the high quality of its meat, the Retinta breed has held great importance in Spanish history and culture for several centuries. One reflection of this can be seen in the painting “Mercurio y Argos” by Velazquez, dated 1659, which features a reclining cow that, according to ethnological analysis carried out by Aparicio Sánchez [5], belongs to the Andalusian or Extremeño Retinta breed, showing the ancient legacy of this unique breed.

The current Retinta denomination was described in the 1960s, when three breeds originating from different ancestral bovine stocks (Retinta, Colorada Extremeña and Rubia Andaluza) were included under the same name. Thus, the breeds Retinta and Colorada Extremeña originated from the red bloodline (Bos taurus Turdenatus), which arrived from Africa, and Rubia Andaluza originated from Bos taurus Aquitanicus, which formed the blond European bloodline [6,7,8]. Thus, it is possible to observe different coat colors within the breed (blond animals, Retinta animals and animals with intermediate degrees of the two coats, see Supplementary Materials Figure S1), which could indicate differences at the productive, morphological and behavioral levels, even under the same breeding pattern [9].

Among the animals with a blond coat, there is a subpopulation called Tamarona, which has been bred in closed conditions since the beginning of the 20th century when the Sixth Marquis of Tamarona bred a herd on the Las Lomas estate, located in the south of the Peninsula (Cadiz, Andalusia, Spain). The Tamarona genetic line was originally selected as working animals for plowing (mainly oxen). This led to the breeding and subsequent selection of larger individuals in order to obtain stronger animals, compared to the rest of the Retinta population, which was destined for meat production [5]. The technification of agriculture has resulted in these animals no longer being used for field work, and their numbers have fallen alarmingly.

The objective of the present study was to determine, using genealogical and genomic approaches, whether the differences between the two genetic lines that the breed originated from have been maintained over time, and whether the Tamarona subpopulation has accumulated enough variation to be considered a distinct line within the Rubia line.

## 2. Materials and Methods

### 2.1. Ethical Statement

This study did not require any of the usual handling/management of animals, since we worked directly with the records provided by the National Association of Retinto Breeders. In the same way, blood samples were collected during routine and mandatory health controls scheduled by the relevant governmental administrations and carried out by official veterinarians under animal welfare standards.

### 2.2. Genealogical Study

The entire pedigree records from the Retinta breed (194,638 individuals registered between 1969 and 2023 by the National Retinta Breeders Association) was employed in this study. Among them, 22,004 individuals were currently active in the date of the study, from which 3735 belonged to the pure Extremeño line, made up of animals with the retina coat (EXT or retinto line), and 2206 to the Rubia Andaluza line consisting of animals with a blond coat (RUB or blond line), within which there were 379 individuals from the Tamarona subpopulation (TAM). The pedigree was extended to include all the available information in the breed database, making a total of 36,695 animals. The remaining undifferentiated animals belonged to livestock with variable degrees of influence of the two main lines of the official breed. The estimation of the effective population size (Ne) via log regression of the individual increase of inbreeding coefficient was calculated with EndogV4.01 [10] software, following the approach by Pérez-Enciso (1995) [11], which presented the increase in inbreeding using the formula:

$$\Delta F=\frac{F_{t}-F_{t-1}}{1-F_{t-1}}\approx \frac{b}{1-\left(F_{t}-b\right)}$$

with *F_t_* the average *F* of the reference subpopulation and *b* the regression coefficient of the individual inbreeding coefficients over the equivalent complete generations. The inbreeding, the average relatedness, the mean maximum generation, the completeness of the pedigree and the generational interval (defined by James 1977 [12]) as the average age of parents at the birth of their progeny kept for reproduction), were calculated using Endog V4.01 [10].

### 2.3. Animal Sampling for the Genomic Assays

For the genomic assays, a total of 1030 animals from 88 different herds were selected from the all the available animals on the ACRE database, avoiding close kinships, according to the criteria proposed by the United Nations Food and Agriculture Organization guidelines for the genomic characterization of genetic animal resources [13]. The animals were grouped into 4 populations: the Rubia Andaluza population (RUB), formed by animals with the blond phenotype from different Andalusian herds; the Tamarona population (TAM), a special genetic line of blond animals within the RUB population; the Pure Retinto Extremeño (EXT), consisting in animals from different herds of the Extremadura region with the Retinta coat color; and a mixed group (MIX), made up of animals from different Andalusian and Extremeño herds with different degrees of influence, to which the majority of animals in the breed belonged. Blood samples were collected using EDTA-K3 BD vacutainers™ (BD, Madrid, Spain) by the official technicians of the ACRE. 

### 2.4. Genotyping and Quality Control

Genomic DNA was isolated from blood using the commercial DNA purification kit DNeasy Blood & Tissue Kit (Qiagen, Germantown, MD, USA), following the manufacturers’ protocol. The purity and concentration of the DNA were measured with a Thermo Scientific™ NanoDrop™ One (Thermo Fisher Scientific Inc., Waltham, MA, USA). Samples that passed the quality control (Absorbance ratios of A260/A280 and A260/230 of 1.8 to 2) were genotyped using the Axiom™ Bovine Genotyping v3 Array (Thermo Fisher Scientific Inc, Waltham, MA, USA), including more than 63,000 SNPs. Genotype calls were obtained by analyzing raw data files following the best genotyping practices using the workflow procedure in the Axiom analysis suite package v5.0 [14]. All the samples showed high-quality genotyping results (DQC ≥ 0.82 and individual call rate QC ≥ 0.90) and were therefore included in this study. After that, markers with a minor allele frequency < 0.05 were removed, which left 33,729 variants. Finally, samples with more than 25% missing data were excluded from subsequent analyses, with 1027 individuals being retained.

### 2.5. Genomic Characterization 

The observed (Ho) and expected (He) heterozygosity and the inbreeding coefficients for each population (FIS) were calculated using the dartR package [15]. The inbreeding coefficient (F) and Multilocus heterozygosity were estimated with the “calcdiversity” function of R package Sambar [16]. Principal coordinate analyses (PCoAs) were performed using the function ‘pcoa’ of the R package ape-5.7.1 (Paradis and Schliep, 2018) using Nei’s genetic distance, calculated with the ‘stamppNeisD’ function from the StAMPP-1.6.3 R package [17]. The genetic distances were calculated by applying ordinary least squares (OLS) clustering to the Euclidean distances. To detect the potential admixture origin of individuals, the genetic distances were compared with the pathlengths obtained in the OLS assay [18]. Elevated residual errors from the hierarchical clustering model indicate an admixture origin of individuals. Thereafter, Nei’s genetic distance [19], was calculated with the StAMPP-1.6.3 R package [17] and visualized in a tree using the ape 5.5 package [20] with the Unweighted Pair Group Method with Arithmetic mean (UPGMA). 

In addition, we estimated the probability of adscription of each individual to their populations using a Bayesian model-based clustering algorithm in the Admixture software V1.3 [21]. Before the assay, the SNPs with linkage disequilibrium (r2 > 0.5) were pruned to generate a dataset of 19,342 SNPs. The structure of the populations was inferred from K = 1 to K = 6 in an unsupervised model and the optimal K values were selected according to the minimal cross-entropy value (see Supplementary Material Figure S2).

### 2.6. Identification of Selection Signatures and Genes Involved

To identify selection signatures in each of the main lines, the database was curated to include only pure Retinto Extremeño and Rubia animals, which included the Tamarona subpopulation, of which 466 individuals remained. A Univariable Linear Mixed Model (uvLMM) were employed using GEMMA software v0.98.5 [22], according the following formula:

*y* = *Wα* + *xβ* + *u* + *ε*

where *y* is an n-vector of case-control (or binary disease labels) for n individuals; *W* is an incidence matrix of covariates (fixed effects) including a column of 1s; *α* is a vector of the corresponding coefficients including the intercept; *x* is an n-vector of marker genotypes; β is the effect size of the marker; *u* is an n-vector of random effects u ∼ N(0, λτ − 1K), where τ is the variance of the residual errors; λ is the ratio between two variance components; K is the genomic relationship matrix (estimated from the markers), and ε is the n-vector of errors. In addition, the model included a correction for population stratification based on the first ten eigenvalues as covariates. The data were corrected by population structure and the genomic relationship between individuals. A Manhattan plot and quantile-quantile (QQ) plot (see Supplementary Material Figure S3) were obtained using the qqman R package [23] in the R studio environment (RStudio Team 2020). Based on the chromosomal location and relative position according to the *Bos taurus* reference genome (GCF_000003055.6), each variant associated with a *p*-value above 1.0005 × 10^−7^ were tested to find the candidate genes in 100 Kb windows (50 Kb upstream and 50 Kb downstream from each point), using the application BioMart from the Ensembl repository. Next, the genes were entered in the DAVID database to study the Gene Ontology [24,25].

## 3. Results

### 3.1. Genealogical Study

The analysis of the entire pedigree of the Retinta breed showed than cows had a higher average age than bulls at the birth of their progeny kept for reproduction in both male and female calves (Table 1). In the case of the Tamarona line, the generational interval among the breeding cows was greater than that shown by the cows from the other lines.

The different parameters obtained from the pedigree analysis are shown in Table 2. The Ne estimation calculated from the pedigree records in the two genetic lines (EXT: 34.47 and RUB: 28.38) were much lower than the value for the whole breed (70.65). Despite its low census, the Tamarona subpopulation showed a slightly higher level of Ne than the Rubia line. Inbreeding values ranged from 0.046 to 0.091, while the mean average coefficient of relatedness showed a minimal value of 0.020 in the whole breed and 0.109 in Tamarona animals. The mean maximum generation ranged between 10.54 to 6.55, while the mean of completeness ranged between 3.89 to 2.97.

### 3.2. Genomic Diversity

**Figure 1:**
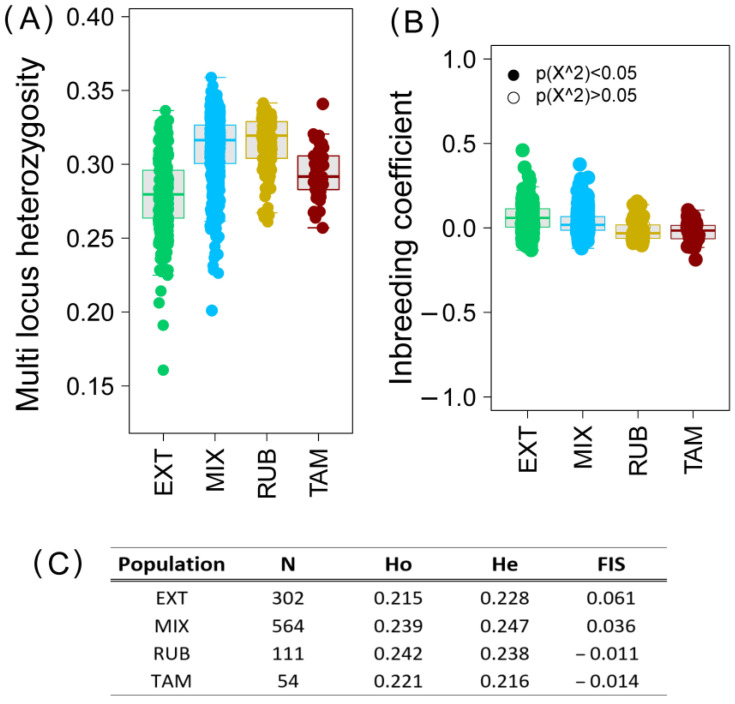
Genomic diversity of the Retinto breed classified by genetic lines. (**A**) Multi-locus heterozygosity at individual level. (**B**) Inbreeding coefficient at individual level. (**C**) Observed (Ho), expected (He) heterozygosity and the inbreeding coefficient (FIS) for each population (N, individuals of each group). In green, EXT (Pure Extremeño animals); in blue, MIX (Mixed group); in yellow, RUB (Rubia population); and in red, TAM (Tamarona genetic line).

Figure 1 shows the individual genomic diversity as multi-locus heterozygosity (Figure 1A) and inbreeding coefficient (Figure 1B). The observed and expected overall heterozigosity inbreeding coefficient of the different Retinto population analyzed is shown in Figure 1C. Ho ranged between 0.215 (EXT) and 0.242 (RUB) while the He ranged between 0.216 (TAM) to 0.247 (MIX). The observed heterozygosity was higher than the expected heterozygosity in the RUB and TAM populations, in contrast to the results found in the EXT and MIX groups, where the Ho levels were lower than the He levels. The inbreeding coefficient values per group were similar in the RUB and TAM populations (−0.011 and −0.014 respectively), which had the highest levels of inbreeding, while the lowest appeared in the EXT population, where the animals showed the highest level of genomic variability.

### 3.3. Population Structure and Genetic Differentiation

**Figure 2:**
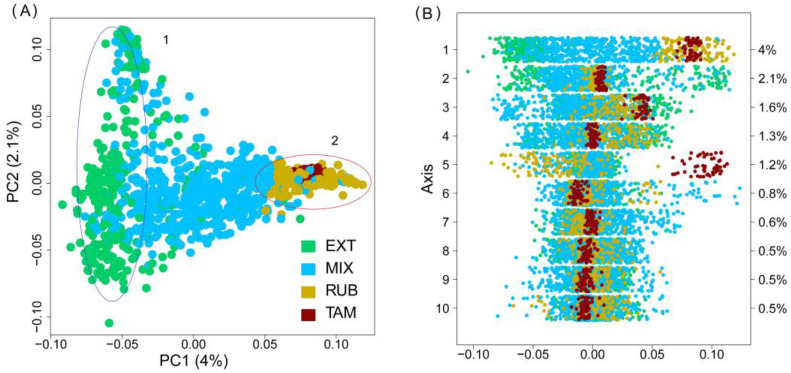
Genetic structure at individual level. (**A**) Principal Coordinates Analysis of the population in the Retino breed. Blue ellipse (1) corresponds with the Pure Extremeño line, while red ellipse (2) corresponds with animals with a blond coat. (**B**) Scatterplot showing the first two ordination axes according to the PCoA. Population: EXT (Pure Extremeño animals), MIX (Mixed group), RUB (Rubia population) and TAM (Tamarona genetic line).

The Principal Coordinates Analyses (PCoA) calculated for the Retinto breed showed a specific clustering for each group of animals according to their initial population, which overlapped at the borders (Figure 2A). The first component, which captured 4% of the variability, grouped animals with a blond coat (RUB and TAM) opposite the animals from EXT, while those from the MIX group were located between them. The second PC axis (accounting for 2.1% of the total variance) showed two principal groups within the EXT line. Tamarona genetic line animals showed a homogeneous aggregate group within the group of blond animals which can be seen on the PC1 to PC4 axis and were clustered in an isolated group on the PC5 axis (Figure 2A,B).

**Figure 3:**
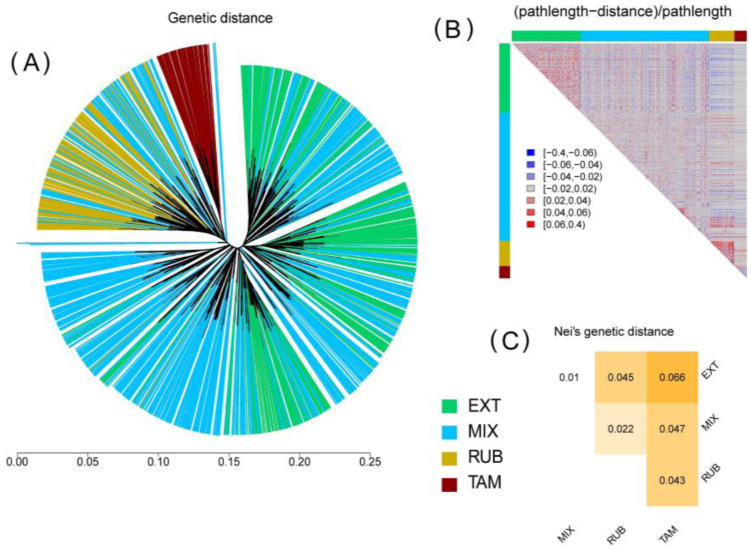
(**A**) Dendrogram depicting Euclidean genetic distances between individuals. (**B**) Residual errors of the OLS clustering model. Red means attraction: the actual distances between individuals are smaller than those suggested by the dendrogram. Blue means repulsion: the actual distances between individuals are greater than those suggested by the dendrogram. (**C**) Nei’s Genetics distances between each population analyzed of the Retinto breed. Population: EXT (Pure Extremeño animals), MIX (Mixed group), RUB (Rubia population) and TAM (Tamarona genetic line).

The dendrogram between individuals suggests a heterogeneous population with different degrees of admixture (Figure 3A). The figure shows the heatmap revealing the differences between the pathlengths and the actual genetic distances. Individuals with a blond coat generally were grouped in the same branch, especially in the case of the Tamarona line. Although some individuals from the MIX group were located in the same branch as the RUB line, the majority cohort of animals from the MIX group were distributed among the Retinta line. Genetic distances between populations showed low values and ranged between 0.01 to 0.066, with the lowest distance between the EXT and MIX populations and the highest distance between TAM and EXT animals (Figure 3C). The heatmap of the residual errors of OLS (Figure 3B) shows how the genetic distances between the RUB and the EXT populations are underestimated according to the current situation. The results showed a greater influence of the EXT line in the Mixed group than that evidenced in the blond animals. Contrary to what one might predict, there was a correct estimation of genetic distances for the Tamarona line with respect to the RUB line, except in some individuals with less genetic distance than that theoretically calculated.

**Figure 4:**
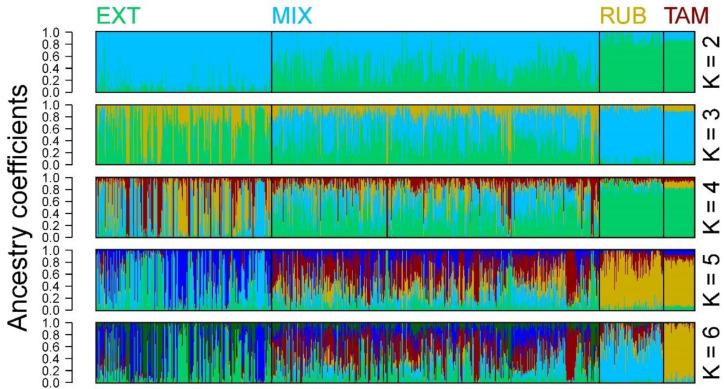
Ancestry coefficients barplot. Figure shows coefficients for K values ranging between 2 and 6 ancestral population groupings. EXT (Pure Extremeño animals), MIX (Mixture group), RUB (Rubia population) and TAM (Tamarona genetic line). Each individual is represented by a single vertical line broken into K colored segments, with lengths proportional to each of the K inferred clusters.

The structure of the population though the admixture analysis showed at K = 2 a clear differentiation between the EXT and the blond animals (RUB and TAM), while the animals belonging to the MIX population showed a variable degree of adscription to both populations (Figure 4). At K = 4 and 5, the blond animals still kept together as a uniform group while the EXT and MIX animals appeared as a heterogeneous, mixed group. At K = 6 (minimal cross entropy level), the Tamarona animals were detected as a subpopulation within the blond line.

### 3.4. Identification of Selection Signatures and Genes Involved

**Figure 5:**
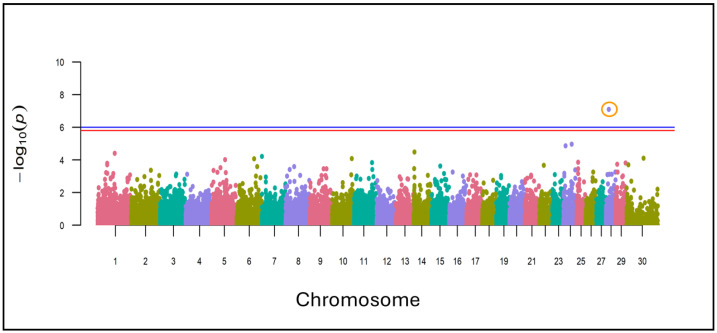
Manhattan plots of the associated DNA points from the genomic regions that have undergone selection events in the Blond and Extremeño groups. Horizontal blue line indicates the suggestive line and red line indicates the genome-wide significance. Orange circles mark the SNPs over the suggestive line.

The analysis showed the presence of an SNP (G/A) located on chromosome 28 at position 13,802,852 with a high level of significance (*p*-value = 7.986442 × 10^−8^) and differentiated frequencies between the blond and pure Extremeño animals (Figure 5). Within the 100 Kb window from the SNP were the HNRNPF, FXYD4, BICC1, RASGEF1A, CSGALNACT2, and RET genes.

## 4. Discussion

Spain is a country in which cattle production has great importance and economic impact, with beef cattle accounting for 15% of the final production in Spanish agriculture. This means that in 2021, it generated approximately 3200 million euros, making it the third country in Europe with the greatest impact, after France and Germany [2].

The Retinta breed, included in the Official Catalog of Livestock Breeds of Spain as a promotion breed, has an approximate national census of 200,000 cows, of which over 10% are registered in its Stud Book [2]. These data make the Retinta the second autochthonous Spanish breed with the highest census in all of Spain and the predominant breed in the southwestern peninsular quadrant, with 96% of its animals in the communities of Extremadura and Andalusia [26]. The level of complete generations of the pedigree is higher than that of other native Spanish breeds, which gives great reliability to the results obtained [27].

The current census of the breed, together with the effective size, shows that the breed has a stable population. The effective size is greater in the whole breed because, in addition to the two pure genetic lines, there are a large number of undifferentiated or mixed herds that show different degrees of influence from the other lines and show great genetic variability. Between the two genetic lines, it was observed that the EXT line presented a higher Ne than the RUB line due to its significantly higher census. However, although the census of the Tamarona subpopulation is much lower than that of the Rubia line from which it derived, the Ne remained higher due to the management system. Tamarona animals are selected by morphotype (including coat color), applying the criterion of avoiding inbreeding by direct crossbreeding to ensure variability (Table 2). In any case, in contrast to the entire breed, the Ne of each line (under the 50 individuals) indicates that the population could suffer an important genetic decrease in variability [28], since the breeding program focuses on the genetic connection between different herds regardless of the genetic line of the animals. In this context, breeders of pure lines should continue to maintain practices that avoid the uncontrolled increase of inbreeding and should increase genetic variability as much as possible.

The Retinta is a breed that has been selected for its rusticity and marked maternal character, and which shows a high reproductive efficiency determined by its genetics [29]. This is reflected in the inter-generational interval, which was greater in females than in males (Table 1), due firstly to the fact that bulls have a shorter productive life, so they are often used earlier in reproduction than cows, and secondly, that the cows are selected for the next breeding window depending on the year of birth and therefore the animal’s maturity and morphological constitution; otherwise, the breeder will wait for the next one.

Considering that the age of first calving is 33.9 months in the breed, the generational interval is approximately between 33.7–34.9 and 58.7–59 months higher in bulls and cows, respectively. This could indicate that animals destined for breeding are generally born from second gestations (for bulls) and second and/or third gestations (for cows). This lack of precocity has been reported to be common in breeds of great longevity where cows that start their reproductive life later are less likely to be culled for productive reasons [30]. Breeders of Tamarona animals, due the exhaustive controls to avoid inbreeding, takes this lack of precocity to the extreme and uses cows in reproduction at a very late age (Table 1).

Since genealogical information is probabilistic, in recent years it is being replaced by genomic approaches that provide information on the actual frequency of alleles and the accumulated differences between different populations due to their transmission. The level of genetic variability of the Retinta breed has been analyzed in previous studies obtaining heterozygosity values of 0.304 [31]. However, the overall heterozygosity levels found in the different Retinto populations in the present work have been lower than those previously found. This might point to a loss of variability that has occurred in recent years within the breed. However, the methodology used in the Cañas-Alvarez work was the high-density SNP chip (BovineHD BeadChip (Illumina Inc., San Diego, CA, USA), with 777,962 SNPs) instead of the medium density SNP chip used in the present work, and it is possible that the number and type of markers used in the two platforms could have influenced this specific value. The results showed that the Extremeño animals and those from the mixed herds showed levels of observed heterozygosity lower than expected, pointing to a loss of genetic variability, but within these populations, the individuals were very heterogeneous both in terms of multilocus heterozigosity and inbreeding coefficient, reflecting a great richness of genetic variants (Figure 1A,B). The blond animals (RUB and TAM) showed the opposite, with Ho higher than He, although their individual variability was evident, despite their small size, and due to the special importance given to them by the breeders, who manage them in a very effective way to avoid genetic drift and lower inbreeding (Figure 1A–C).

The genetic distances between the different groups analyzed were not very high (Figure 3C). Even so, the degree of genetic differentiation between the Blond and the Extremeño lines surpasses levels typically observed in other Spanish breeds extensively raised for meat production, such as the Asturiana de los Valles, Avileña, or Rubia Gallega, among others. Similar genetic disparities at the breed level are also evident between animals from the Tamarona subpopulation and those belonging to the Rubia line. In addition, the results of the PCA, together with the analysis of the population structure, showed that there were clear differences between the groups employed (Figure 2 and Figure 4). In this way, it is clear that the blond-coated animals (RUB and TAM) are quite differentiated from the pure Extremeño animals, possible to observe in the middle the group of animals from mixed herds can be observed in the middle. Within the MIX group, the herds with more Andalusian influence were genetically closer to the blond animals, while the herds with low Andalusian influence were more like the pure Retinto animals. The level of admixture of the undifferentiated herds show the clear preferences of the breeders to enhance the characteristics of the pure Retinto line, perhaps due to the differences in the behavior between the two pure lines: while Retinto animals are usually more docile and easier to manage, blond animals are less popular among breeders because of their drier character.

One of the characteristics of blond animals is that they tend to be larger than Retinto animals, especially in the case of the Tamarona subpopulation [9]. The search for selection footprints has allowed us to detect three genes that could be related to body size and growth. The *BICC1* gene (BicC Family RNA Binding Protein (1) has been implicated in developmental processes and organ size regulation in other species [32,33], and there is some evidence suggesting that genetic variations influence body size and weight in cattle [34]. The *CSGALNACT2* gene (Chondroitin Sulfate N-Acetylgalactosaminyltransferase (2) is involved in the biosynthesis of chondroitin sulfate, an important component of cartilage [35,36]: genetic variations in this gene can influence skeletal development and, consequently, body size and weight in cattle. [37]. Finally, the *RET* (Ret Proto-Oncogene) is involved in cell proliferation and differentiation [38,39] and is associated with body size and growth traits in cattle [40]. These results confirm the trend among breeders of the blond line towards selecting for larger animals.

As expected, the animals in the TAM line were located within the group of blond-coated animals. Even so, they presented sufficient genetic differences to be considered a special group showing the lowest level of admixture (Figure 4), and the results obtained are consistent with the history of the breed. In fact, the blond-coated animals were considered a separate breed until 60 years ago, so there has not been time for a generalized mixing between individuals of the two original breeds, maybe since the blond character (phenotypically detectable in situ) was attractive to some breeders, which may have led to a more straightforward, direct selection process.

The genomic approach supports the idea that, although both genetic lines have been conserved, the whole population has been severely influence by the Retinta line which gives the breed its name. The blond line has exercised less influence in general, especially in the Tamarona subpopulation that has been totally free of external influences due to its closed system of management. The fact that these great differences between groups of animals originating from different breeds have been maintained should raise the question of whether it is in our interest to conserve and protect this genetic wealth. However, we feel it is worth preserving the line of blond animals because of the phenotypic particularities that make them different and unique. Within this line, the Tamarona subpopulation has maintained the highest degree of purity and has even accumulated enough changes to be considered a different genetic line. These variations increase the genetic richness of this autochthonous livestock which, in times of scarcity, drought or adverse situations, will always be the best adapted to local environments. The alternatives we have for future conservation lie in attempting to have it recognized either as a breed, or as a lineage.

## 5. Conclusions

In conclusion, the Retinta and Blond subpopulation continue to uphold their pure genetic lines with clear genealogical and genomic differences, although the influence of the Retinto line in the breed is higher and more predominant. Although breeders prefer the Retinto coat, the blond line is perpetuated in some herds by avoiding further external influence due the ease of selecting by coat color. In addition, within the blond population, the Tamarona subpopulation has accumulated enough genomic differences to be considered as a new genetic line of the breed.

## Figures and Tables

**Table 1:** Generational interval in the Retinto breed and its genetic lines.

Population	Breeding Animal	Offspring	N	GI
Global	Bull	Male	1497	5.85
		Female	12,663	5.63
	Cow	Male	1497	7.77
		Female	12,732	7.50
EXT	Bull	Male	425	5.62
		Female	2932	5.42
	Cow	Male	425	7.70
		Female	2933	7.38
RUB	Bull	Male	462	5.69
		Female	2021	5.75
	Cow	Male	462	7.24
		Female	2019	7.27
TAM	Bull	Male	6	5.79
		Female	120	5.72
	Cow	Male	6	8.27
		Female	120	8.72

N: individuals used for the estimations. GI: generational interval. Population employed: Global (the whole Retinto breed population); EXT: individuals from the pure Retinto line; RUB (individuals from the blond line); TAM (individuals from the Tamarona subpopulation).

**Table 2:** Main demographic parameters obtained from the genealogical analysis of the populations of the Retinta breed analyzed.

	Global	EXT	RUB	TAM
N (Ne)	30,448	6169	2649	379
Ne	70.65	34.47	28.38	31.35
N (F, AR)	36,695	8341	4798	970
F	0.062	0.091	0.046	0.056
AR	0.020	0.057	0.027	0.109
MG	9.78	10.54	7.91	6.55
CG	3.22	3.43	2.97	3.89

N (Ne): individuals used for Ne calculation; Ne: effective size obtained from log regression on birth date; N (F, AR): individual used for F and AR calculation; F: mean inbreeding; AR: mean average coefficient of Relatedness; MG: Mean Maximum Generations; CG: Mean Complete Generations. Population employed: Global (the whole Retinto breed population); EXT (individuals from the pure Retinto line); RUB (individuals from the blond line); TAM (individuals from the Tamarona subpopulation).

## Data Availability

All data contained within the article.

## Supplementary Materials

The following supporting information can be downloaded at: https://www.mdpi.com/article/10.3390/vetsci11060247/s1, Figure S1: Morphotype of the animals of each ancestral line within the Retinto breed; Figure S2: Cross-entropy value of the population analysis for K1 to K6. Figure S3: Quantile-quantile (QQ) plot of the associated DNA region to selection signatures.
