# Supplementary material for: Genomic Diversity of the Retinta Breed Derived from Two Ancestral Bovine Lineages
Source: Vet Sci. 2024 May 30;11(6):247. doi: 10.3390/vetsci11060247 (PMC11209511; doi:10.3390/vetsci11060247)

Supplementary Materials

Figure S1: Morphotype of the animals of each ancestral line within the Retinto breed. Cow (A) and Bull (B) of the Pure extremeño line; Cow (C) and Bull (D) of the Rubia Andaluza line.

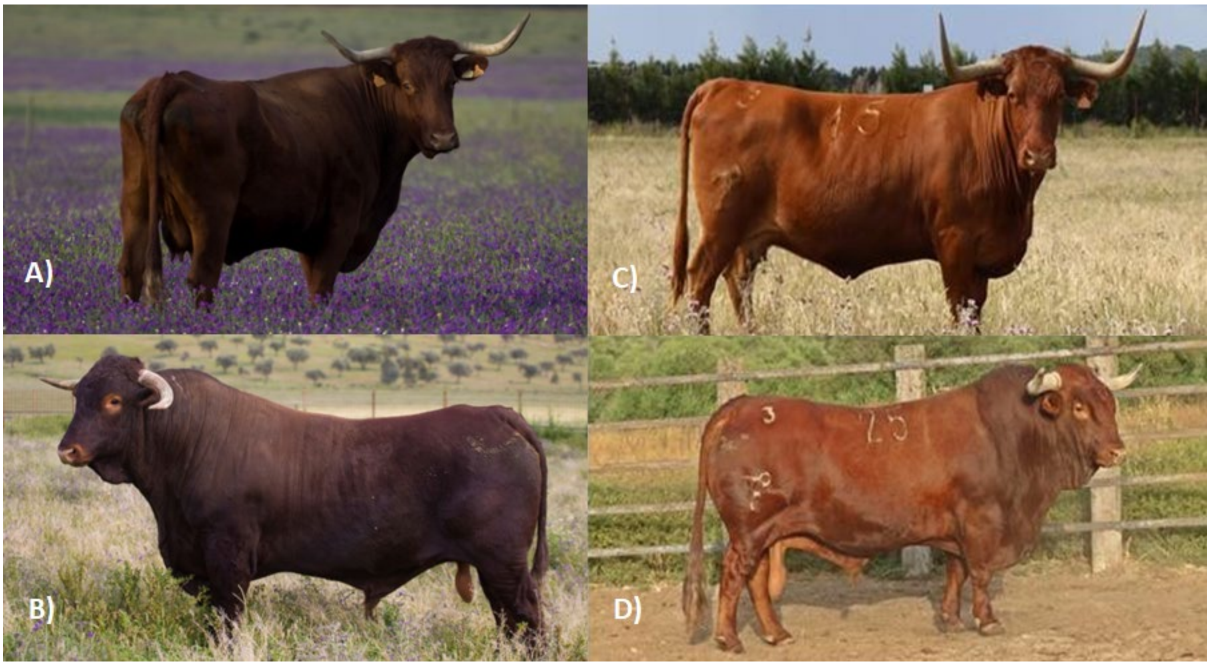

Figure S2: Cross-entropy value of the population analyse for K1 to K6

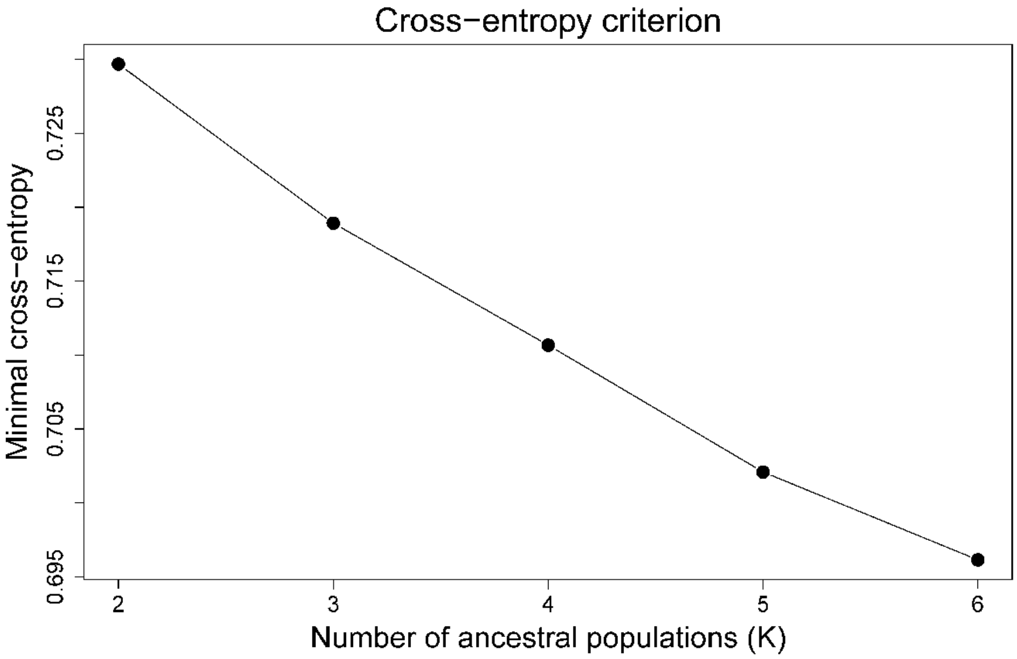

Figure S3: Quantile-quantile (QQ) plot of the associated DNA region to selection signatures.

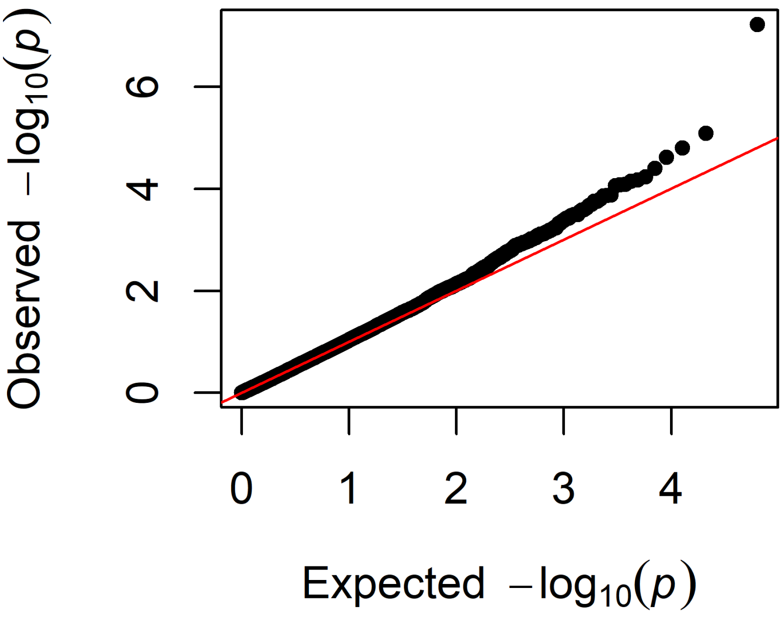

Supplement: Supplementary file 1 [file vetsci-11-00247-s001.zip › vetsci-2997713-supplementary.pdf]
